# Novel *Ex Vivo* Models of Epithelial Ovarian Cancer: The Future of Biomarker and Therapeutic Research

**DOI:** 10.3389/fonc.2022.837233

**Published:** 2022-03-25

**Authors:** James Clark, Christina Fotopoulou, Paula Cunnea, Jonathan Krell

**Affiliations:** ^1^ Division of Cancer, Department of Surgery and Cancer, Faculty of Medicine, Imperial College London, London, United Kingdom; ^2^ West London Gynaecological Cancer Centre, Imperial College NHS Trust, London, United Kingdom

**Keywords:** epithelial ovarian cancer, patient-derived organoids, explant tumour slice, biomarkers, therapy

## Abstract

Epithelial ovarian cancer (EOC) is a heterogenous disease associated with variations in presentation, pathology and prognosis. Advanced EOC is typified by frequent relapse and a historical 5-year survival of less than 30% despite improvements in surgical and systemic treatment. The advent of next generation sequencing has led to notable advances in the field of personalised medicine for many cancer types. Success in achieving cure in advanced EOC has however been limited, although significant prolongation of survival has been demonstrated. Development of novel research platforms is therefore necessary to address the rapidly advancing field of early diagnostics and therapeutics, whilst also acknowledging the significant tumour heterogeneity associated with EOC. Within available tumour models, patient-derived organoids (PDO) and explant tumour slices have demonstrated particular promise as novel *ex vivo* systems to model different cancer types including ovarian cancer. PDOs are organ specific 3D tumour cultures that can accurately represent the histology and genomics of their native tumour, as well as offer the possibility as models for pharmaceutical drug testing platforms, offering timing advantages and potential use as prospective personalised models to guide clinical decision-making. Such applications could maximise the benefit of drug treatments to patients on an individual level whilst minimising use of less effective, yet toxic, therapies. PDOs are likely to play a greater role in both academic research and drug development in the future and have the potential to revolutionise future patient treatment and clinical trial pathways. Similarly, *ex vivo* tumour slices or explants have also shown recent renewed promise in their ability to provide a fast, specific, platform for drug testing that accurately represents *in vivo* tumour response. Tumour explants retain tissue architecture, and thus incorporate the majority of tumour microenvironment making them an attractive method to re-capitulate *in vivo* conditions, again with significant timing and personalisation of treatment advantages for patients. This review will discuss the current treatment landscape and research models for EOC, their development and new advances towards the discovery of novel biomarkers or combinational therapeutic strategies to increase treatment options for women with ovarian cancer.

## 1 Introduction

Ovarian cancer is a spectrum of different clinical and pathological entities with variable presentations and behaviour. Epithelial Ovarian Cancer (EOC), of which the most common subtype is serous, comprises approximately 90% of cases (1). Other epithelial subtypes include endometrioid (EM), clear cell (CC) and mucinous (MC) (2). High-grade serous ovarian cancer (HGSOC) is responsible for the majority of EOC deaths (1) and most patients present with advanced disease, which is typified by frequent relapse and a 5-year survival of less than 30% despite treatment (3). Although it is termed HGSOC, the secretory epithelial cells of the distal fallopian tube are thought to be the most common progenitors for this subtype (4, 5).

HGSOC is a genomically diverse cancer, displaying significant genomic instability and intra-tumoural heterogeneity. TP53 mutations are almost ubiquitous (96%), BRCA1/2 germline and somatic mutations are observed in 22% of tumours and approximately half of cases display deficiencies in homologous recombination (6). HGSOC also demonstrates extensive copy number aberrations e.g. CCNE1 amplification (7), which has been associated with worse outcomes and chemoresistance (8). While HGSOC has the highest proportion of copy number alterations (CNA), CNA are also observed to a lesser extent in other ovarian cancer subtypes (9). EM and CC ovarian cancers are typically associated with endometriosis and often harbour ARID1A mutations. Mucinous ovarian cancer typically harbours KRAS or TP53 mutations, or ERBB2 amplification but is much rarer than other subtypes of ovarian cancer (approximately 3%) (10). Many of the presumed mucinous ovarian cancers are in fact metastatic cancers e.g. from gastrointestinal epithelium or endocervical glands (11). Low-grade serous ovarian carcinomas (LGSOC) frequently possess KRAS, ERBB2 and BRAF mutations as well as WNT signalling pathway alterations (7). Epithelial ovarian cancers have very low rates (<2%) of mismatch repair (12) and rates of response to single agent checkpoint inhibition are low (4.1%) (13).

## 2 The Current Ovarian Cancer Diagnostic and Treatment Landscape

High grade serous ovarian cancer typically presents in postmenopausal women with non-specific symptoms such as bloating, abdominal pain, distension, gastrointestinal disturbance or systemic symptoms such as sweats or weight loss. It can also manifest as an incidental finding. Cancer antigen-125 (CA-125) is frequently elevated, and the use of ultrasonography and multimodal cross-sectional imaging (e.g., CT and MRI) is crucial to establish accurate radiological staging prior to treatment decision making.

### 2.1 Surgery

In conjunction with systemic approaches, maximal effort surgery is one of the cornerstones of ovarian cancer treatment. Even in the advanced forms of the disease (i.e., FIGO stage III/IV), there is substantial evidence demonstrating that surgical tumour debulking is significantly associated with prolonged remission and survival, including in those situations of high tumour burden (14, 15). Valid quality of life (QoL) data show that surgical radicality is not associated with long term impairment of QoL scores but instead with marked improvement compared to baseline (16).

Debulking surgery can occur in the primary upfront or interval setting following neoadjuvant chemotherapy depending on the presentation, pathology and pattern of the disease, but also patient related factors such as performance status, fragility scores and patient wishes. Large prospective randomised trials with translational aspect are awaited to clarify the question of timing of surgery in operable patients within a specialised setting (17, 18). Regardless of the timing of surgery however, eligible patients should be directed towards specialised ovarian cancer centres where they can undergo high expert surgery to optimise outcomes. Focussed attempts have been undertaken by the largest gynaecological oncology societies worldwide (such as the European Society of Gynaecologic Oncology), to define and establish surgical quality indicators to homogenise and standardise surgery, towards centralisation of surgical care (19, 20). Furthermore, patients’ education, coaching and thorough informed consent about the associated risks and benefits of surgery are crucial for surgical success and preparation of patients for their treatment pathway (21).

### 2.2 Chemotherapy

Given the high proportion of homologous recombination (HR) deficiencies in HGSOC, treatment development has focused on targeting DNA repair to exploit these defects therapeutically (22). The standard-of-care treatment for advanced disease is conventional platinum-based chemotherapy, usually in combination with paclitaxel. Platinum treatment induces inter- and intra-strand DNA cross-linking with subsequent replicative and transcription arrest in tumour cells (23, 24). This results in apoptosis in a p53 dependent manner (25). This cross-linking is particularly lethal in patients with HR deficiency (HRD), although platinum sensitivity can also occur as a result of defective nucleotide excision repair (26). Of the platinum compounds, carboplatin has been shown to be non-inferior to cisplatin in ovarian cancer but with less toxicity (27, 28), and thus is preferred in EOC treatment.

Paclitaxel works *via* a different mechanism and at a different stage of the cell cycle than platinum compounds. Paclitaxel inhibits cell division through promoting stable microtubule assembly during cell division which prevents depolymerisation and disassembly with subsequent G2/M cell cycle arrest (29, 30) and apoptosis (31). Taxol-induced apoptosis is independent of p53 and instead relies on the MAP kinase pathways ERK and p38 (32).

The combination of carboplatin and paclitaxel has been long established as first line treatment in EOC, whether in the neo-adjuvant, adjuvant or palliative setting (33). However, many patients with advanced disease will subsequently relapse despite treatment, with the time to relapse a negative prognostic indicator for survival and chances of responding to further platinum-based treatment (34–36). Other chemotherapy agents have shown some benefit with their use in EOC including pegylated liposomal doxorubicin (37), topotecan (38) and trabectedin (39). However, given the modest benefits observed with these chemotherapeutic agents, further research has therefore been conducted into other treatments to improve survival for EOC patients.

### 2.3 Targeted Agents

#### 2.3.1 VEGF Inhibitors

Vascular endothelial growth factor (VEGF) plays a key role in angiogenesis, a vital component of tumour growth and metastasis (40, 41). Within EOC, VEGF is thought to contribute to neovascularisation, and the extent of vascularity has been demonstrated to negatively impact disease-free and overall survival (42). Furthermore, VEGF also contributes to ascites production through increased peritoneal permeability (43).

VEGF inhibitors, notably bevacizumab, have been extensively trialled within EOC in an attempt to therapeutically exploit this commonly overexpressed pathway. Bevacizumab (an anti-VEGF monoclonal antibody) was the first targeted therapy approved for EOC (in addition to fallopian tube and primary peritoneal cancer). However, its benefit has not been as clear as that seen with carboplatin and paclitaxel in the first-line setting, with retrospective subgroup analyses identifying only those patients with poor prognosis disease as those who benefit significantly from anti-VEGF treatment (44–46). However, for those with high-risk disease, it has provided a useful addition to the ovarian cancer treatment armamentarium, and bevacizumab has since gained regulatory approval with the European Medicines Agency and the US Food and Drug Administration. Exact conditions and evidence for its use are beyond the scope of this article, but the European Society for Medical Oncology/European Society of Gynaecological Oncology guidelines (47) provide a useful overview.

#### 2.3.2 PARP Inhibitors

More recently ovarian cancer treatment has been revolutionised by the discovery of Poly (ADP-Ribose) Polymerase inhibitors (PARPi). PARPi exert their action through binding to the catalytic site of PARP1, preventing its release from DNA and thereby trapping it in the PARP/DNA nucleoprotein complex (48–50). The trapped PARP1/DNA nucleoprotein complexes prevent progression of the DNA replication forks. In cells with viable homologous recombination, stalled replication forks would be repaired and restarted by HR in the first instance (51). However, in cells with defective HR, stalled replication forks have the potential to degrade into cytotoxic double strand breaks. This leads to genomic instability and subsequent apoptosis of the cell (52). PARPi thereby exhibit “synthetic lethality”. PARPi also result in impaired base excision repair in the context of single strand breaks, and promotion of other DNA damage repair mechanism such as non-homologous end-joining (NHEJ) (53).

BRCA proteins are vital to achieve successful HR, and thus PARP inhibition can exploit synthetic lethality in patients with homozygous BRCA mutations. PARPi are also involved in the protection of stalled replication forks and are likely to have further reaching effects given the roles of PARP1 and PARP2 in transcription, apoptosis and immune function (51). In addition, some patients without germline or somatic BRCA mutations also receive benefit from PARP inhibition consistent with the high prevalence of HRD seen in ovarian cancer (6).

Several PARPi have now been licensed following success in advanced EOC in both first line (54, 55) and relapsed settings (56–58), and they now form an integral part of ovarian cancer treatment. Niraparib has demonstrated benefit irrespective of the presence of proven homologous recombination deficiency (55) indicating that further understanding of the complexities of EOC and PARPi is still required.

### 2.4 Resistance to Treatment

However, not all patients will respond to treatment [up to 25% of patients have platinum refractory disease at presentation (59)], and identifying those patients who will benefit from platinum-based chemotherapy and/or PARPi is difficult and remains a significant unmet clinical need. Additionally, even patients with BRCA1/2 mutations who have initially responded to platinum-based chemotherapy or PARPi invariably develop drug resistance through a variety of mechanisms. In BRCA1/2 mutated tumours treated with platinum-based compounds or PARPi, resistance occurs most commonly by secondary reversion mutations in BRCA1/2 that result in restoration of the open reading frame and a degree of HR function (60–64). Other mechanisms include loss of PARP1 expression (65), loss of BRCA1 methylation (66), restoration of HR through inactivation of DNA repair proteins REV 7 (67) or 53BP1 (68) or initiation of drug efflux (60).

Whilst our understanding of potential resistance mechanisms has improved, the significant genetic diversity of this disease and the variability in treatment responses means that reliable methods are required to identify which patients are likely to benefit from particular treatments. In addition, models that accurately mimic the *in vivo* tumour treatment response are needed for further exploration of the possible indicators of resistance as well as evaluation of future treatment options and biomarkers of response.

## 3 Ovarian Cancer Models

### 3.1 2D EOC Cell Lines

2D cell monolayers comprise primary tumour cell cultures and immortalised cell lines. Primary cell cultures are formed from the mechanical dissociation of tumour cells, or from isolation and culture from ascites (69). They more closely resemble the native tumour but are limited by their finite lifespan, permitting sub-culturing for only a few months at most (69). Secondary immortalized cell lines can arise from primary cell cultures, either spontaneously or through induced transformation to overcome senescence, and form a pure, uncontaminated population of tumour cells that possess limitless replicative potential (69). Cell lines are still widely used in high throughput drug screening for preclinical drug development and have been shown to reflect the genomic diversity of their respective tumours (70, 71) with genotypic and phenotypic properties (such as copy number variants (70), mutations (71), gene (72) and protein (73) expression profiles) of a number of different cell lines well characterised.

However, generation of cell lines, whether primary or immortalised, can be time consuming and difficult to establish - for example, only 1 primary culture out of 156 ovarian tumour cultures spontaneously immortalised in one study (74), or multiple steps (e.g. selective trypsinisation) are required to generate a culture of pure tumour cells (75). Cell lines have little native tissue architecture as a function of their culture as a 2D monolayer of cells, but also endogenous cytokines and other cell signalling molecules are absent (76). Importantly they lack interaction with other cell types, although it is possible to co-culture with e.g. cancer associated fibroblasts (77) or immune cells such as macrophages (78), which form a key aspect of their behaviour *in vivo*. It is important to recognise also that any secondary cell line that has arisen in *ex vivo* culture may not represent a true clonal outgrowth that could have arisen *in vivo*, rather a sub-clone that has adapted particularly well to cell culture conditions.

Furthermore with increasing passages, cell lines often change in culture due to the development of an *in vitro* phenotype with genetic drift and new genetic variations not observed in the original tumour arising (76), along with a loss of gene expression in key cancer signalling pathways (79). Such alterations have been corroborated with gene expression analyses (80, 81). This clonal selection can result in homogeneous cell lines with little or no genotypic or phenotypic resemblance to the original tissue, particularly important given the intra-tumoural heterogeneity present in many cancers, notably HGSOC (82). This can lead to an unreliable or unpredictable model that does not accurately represent *in vivo* behaviour. Extensive profiling studies of several well-known EOC cell line models in recent years have demonstrated that certain commonly used lines purported to be particular histological subtypes of ovarian cancer, were found to have different histologies from that previously reported (83, 84).

Cell lines also are susceptible to factors (e.g. cell density, media change) that can influence cell metabolism, which can have a knock-on effect on cellular responses to drug treatments (85). Therefore, whilst cell lines continue to be essential for the establishment of preliminary efficacy, correlation with *in vivo* responses is difficult. While attempts have been made to use primary cell cultures from fresh tumour tissues to personalise tumour cell cultures rather than immortalised cell lines, extended passaging of primary cells can frequently lead to cellular senescence and/or rapid accumulation of chromosomal instability with changes in cell morphology and subsequent cell death (86, 87).

### 3.2 3D EOC Models

#### 3.2.1 Spheroids

Spheroids were initially described in 1971 following culture of cancer cell lines in non-adherent conditions (88). They are three-dimensional multicellular aggregates of tumour cells that can be formed from immortalised cell lines or primary cells and generated with or without a matrix scaffold. Spheroids can be formed from single cells or aggregates of cells, and in general those cells with stem cell-like properties are enriched in spheroid culture in the presence of growth supplements (89).

Use of spheroids as a culture model in EOC is inherently attractive as they are commonly found in ascites (90), and there are similarities observed between spheroids and the EOC cell aggregation in ascites (89, 90). Use of ascitic fluid does have its limitations compared to use of solid tissue e.g., insufficient cellular material for analysis or unavoidable selection of cells during the filtration process with removal of TME components. However, it can be easy to access and also collected numerous times as a therapeutic procedure for patients whilst also potentially permitting contemporaneous sensitivity and resistance analysis.

Spheroids have also been developed from EOC tumours (91, 92) and were largely used for drug sensitivity and resistance analyses given the fact that malignant cells with a stem cell-like phenotype are thought to be responsible for drug resistance (93). Spheroids can also be used in patient derived xenograft (PDX) models. However, they can be difficult to generate, and normal epithelial cells do not grow well in spheroid culture thereby precluding any platform control comparisons (89). They also lack the organ specific complexity seen in patient derived organoids (PDOs) (94). Short-term PDOs have however been successfully grown from ovarian multicellular spheroids (95).

#### 3.2.2 Patient Derived Organoids (PDOs)

PDOs are organ specific 3D structures derived from primary tumour cells or human stem cells, that have now been established for EOC (10, 96–101). They are quicker, cheaper and easier to generate than PDX mouse models (96) and have been shown to have more accurate genotype–drug sensitivity correlations than seen with 2D cultures (97). They require less starting tumour material than PDX models and have a higher success rate for propagation (10). Compared to spheroids, PDOs have a higher degree of complexity, display a greater resemblance to the organ from which they originate, and permit growth of normal and pre-cancerous cells (89).

**Figure 1:**
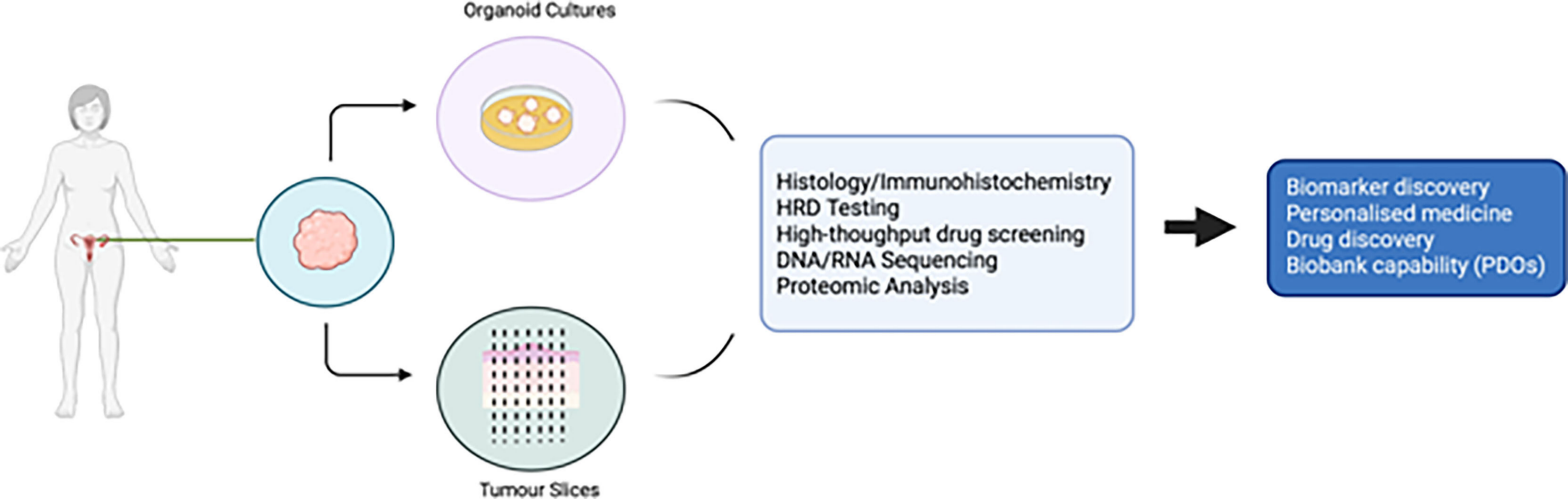
Potential uses of patient derived organoids and tumour slices in ovarian cancer research.

PDO technology lies in the use of a combination of growth factors and small molecules used in combination with a basement membrane mimic (e.g., basement membrane extract such as Matrigel) (98) in an attempt to represent a long-term growth environment. PDOs permit evaluation of clonal heterogeneity in tumours (96) and the rapid timeframe of generation results in less *ex vivo* selection than that seen in cell lines or PDX models. They are relatively easy to manipulate and generate functional assays on which to test drug treatments. A summary of the advantages and disadvantages of different EOC models is presented in **Table 1**, and the potential uses of PDOs and explants is presented in **Figure 1**.

**Table 1:** Advantages and disadvantages of different Ovarian Cancer 3D models.

Model type	Advantages	Disadvantages
**Spheroid**	- Can be formed from immortalised cell lines - Long term expansion possible - Low-High throughput drug screening - Similar genomics/phenotypes to primary tumour if established with primary cells - 3D culture more accurately representing *in vivo* conditions - Can be genetically manipulated - Can be transplanted into PDX models - Facilitate cell:cell and cell:matrix interactions - Promote expression of stemness transcriptional factors	- No TME - Specific spheroid model for a particular tissue/organ required which can be time consuming to establish - Diffusion gradient with increased spheroid size with hypoxic/nutrient deficient core - Less organ specificity and complexity than organoids - No stromal interactions
**PDX Model**	- *In vivo* - Re-capitulation of TME - High heterogeneity possible - Availability of drug sensitive and resistance models - Can assess dose limiting organ toxicity	- Adaption to murine environment - Time consuming, costly, labour intensive and ethical issues - Variable success rate – more aggressive tumours transplant better - Difficult genetic manipulation - Only low throughput drug screening
**Organoid**	- Long term expansion possible - Low-High throughput drug screening - Similar genomics/phenotypes to primary tumour - Organ specificity - Can be genetically manipulated - Short timeframe of generation once model generated - 3D culture more accurately representing *in vivo* conditions than immortalised spheroids - Facilitate cell:cell and cell:matrix interactions - Suitable for different tissue subtypes, and can grow healthy or malignant tissue - Can be transplanted into PDX models - Promote expression of stemness transcriptional factors	- Rely on self-organisation capabilities of cancer cells - No stromal interaction, though some co-cultures possible - Accumulation of mutations with increasing passages - Specific organoid model required for a particular tissue/organ which can be time consuming to establish - Diffusion gradient with increased organoid size with hypoxic/nutrient deficient core - Difficult to re-create some aspects of the TME such as mechanical stress and interstitial fluid flow - Variable derivation rate
**Tumour Explants**	- Retain tissue architecture with majority of TME components represented - Permits evaluation of tumour cell behaviour within their own ECM and surrounding microenvironment - Short generation time and fast readout of results - Low-High throughput drug screening - Suitable for different cancer types - Assays can be performed on factors released into growth media	- No self-renewing capabilities – long term expansion not possible - Require reasonable amount of tumour tissue to generate successfully - Practical difficulties with tumour slicing – success rate tumour and operator dependent - Short duration of cell viability
**Organotypic Models**	- Can aid understanding of tissue invasion and metastasis in HGSOC - Incorporates some TME components with more recent models incorporating more diverse components - Self-renewing capabilities - Facilitate cell:cell and cell:matrix interactions - High throughput drug screening possible -	- High throughput drug screening possible - Not all TME elements incorporated - Time consuming to generate - Rely on an artificial ECM as with other models -

**Figure 2:**
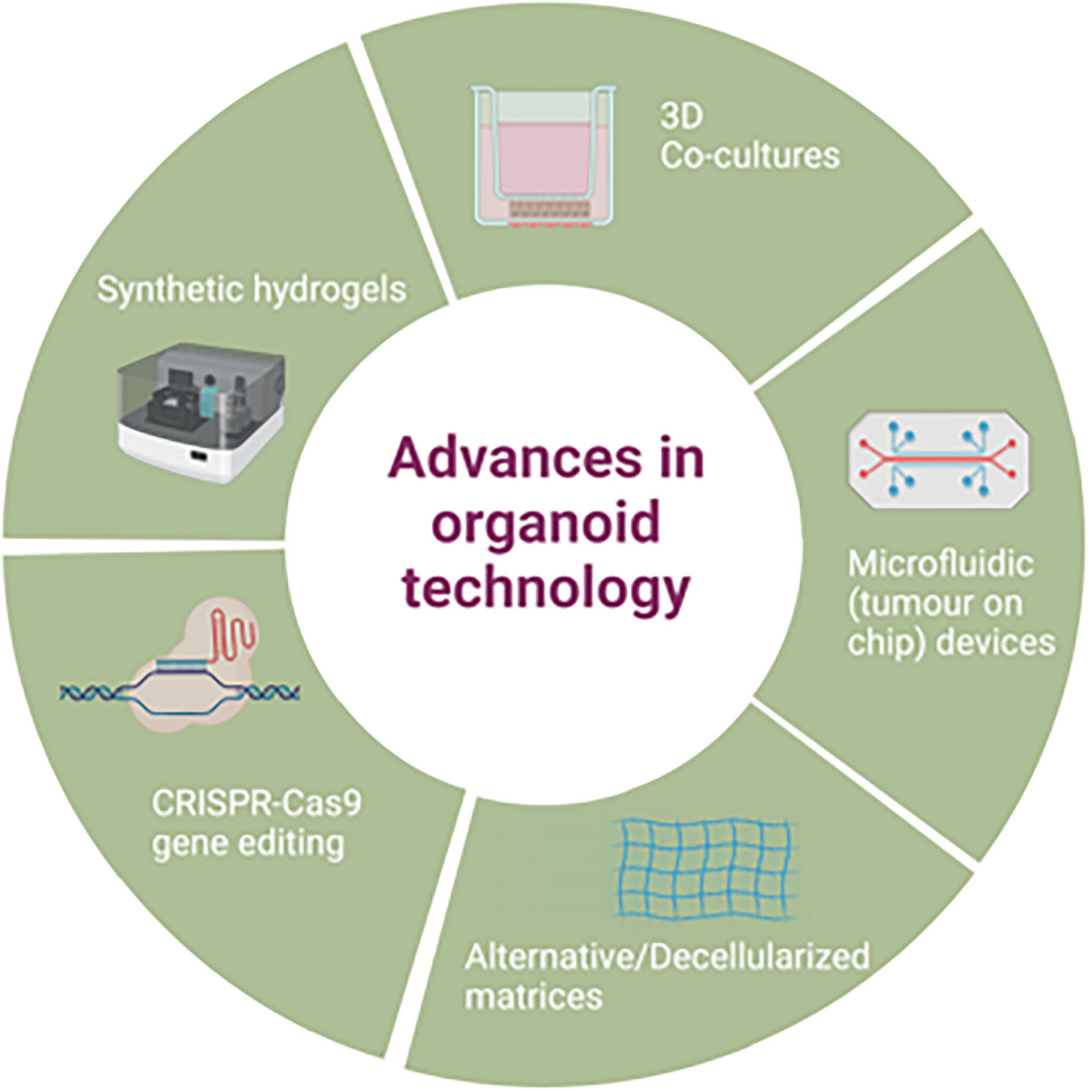
Advances in patient derived organoid technology.

Improvements on the original organoid methodology have also been attempted e.g. use of a mini ring method in comparison to traditional drop seeding, to quickly generate organoids from a small number of cells to use for a rapid turnaround for high throughput drug screening (102), though this approach has not yet been widely adopted. Further advances in organoid applications are discussed in section 5, as well as demonstrated in **Figure 2**.

#### 3.2.3 Patient Derived Xenografts (PDX)

PDX models have been established in a variety of different cancer types including EOC (103). PDX models are generated from the injection of tumour cells or tissue into immunocompromised or humanised mice. This can be done heterotopically (usually subcutaneously for ease of measurement) or orthotopically following dissection +/- digestion of the original tumour. Alternatively, cell lines can be used for generation of xenografts. Whilst subcutaneous administration is often favoured due to its ease, it is regarded as inferior to orthotopic transplantation in terms of clinical correlation, metastatic potential and TME similarity to host tumour (104). Intraperitoneal PDX models have also been established with favourable engraftment success rates (103), although orthotopic injection is still considered the gold standard for modelling HGSOC (104).

PDX models more accurately represent the three-dimensional TME than cell line xenografts by better retaining tumour architecture and containing components of the original tumour stroma, vasculature and immune cells (98). They have been shown to correlate morphologically and histologically with original tumours (105), can exhibit corresponding pathological single nucleotide polymorphisms (106) and exhibit comparable gene expression profiles (107).

However, generation of PDXs generally require a significant amount of surgical specimen and have limited engraftment success rates which are variable and tumour specific (108). They are also time consuming, taking up to 8 months to develop (109), with limited capability for extensive testing of different drug therapies. In some cases, this timeframe may be beyond that of the patient’s prognosis. PDX models are not suited for genetic manipulation or large-scale drug screening and undergo mouse specific tumour evolution that can lead to fundamental differences genotypically and phenotypically from the original tumour (110, 111). For example, increasing number of PDX passages has been shown to lead to increased accumulation of mutations and higher growth rates of engrafted tumour, with tumour grade correlated with increasing PDX passage number, suggesting clonal selection (112). Human stroma also becomes replaced with mouse stroma (113) thereby reducing the *in vivo* similarity with the tumour-TME interface as well as differences in pharmacodynamics and pharmacokinetics between human and mouse (111). Mouse fibroblasts may even outgrow co-injected CAFs which play a significant role in tumour growth and progression (114).

In addition, the type of optical imaging required in PDX models presents additional complications and cost. Bioluminescence imaging requires genetic manipulation of cancer cells to incorporate bioluminescent gene expression with further selection required of expressing cells (104, 115). CT/PET-CT is time consuming, expensive and low throughput (104). However, alternative fluorescence imaging methods have been developed such as fluorine-18-labelled trimethylacetate-based radiotracer to aid *in vivo* tumour visualisation (116).

#### 3.2.4 Tumour Slices/Explants

Primary tumour explant or slice cultures are generated directly from freshly resected tumours. As such they maintain tissue architecture and spatial organisation, much of the TME, and can depict the inherent intra-tumoural heterogeneity of the tumour (117, 118). This permits evaluation of tumour cell behaviour within their own ECM and surrounding microenvironment (119). Fluorescence-based and live multiplex imaging of explants can also enable visualisation of the abundance of TME components (such as cancer cells, immune cells, blood and lymphatic vasculature) *in situ.* Explants have demonstrated representative cellular populations compared to *in vivo* tumour, and also alteration in the abundance and phenotype of immune cells in response to cytokine stimulation and immune checkpoint inhibition (119). This ability to accurately represent the immune aspect of the TME is important as immunotherapy requires an intact TME to function (120)

Furthermore, explant cultures can be generated quickly with minimal manipulation of the tissue beyond dissection and culture, and therefore drug sensitivity and resistance assays can be assimilated and analysed with minimal delay. Such drug screening can be medium-high throughput providing sufficient sample size and can generate a personalised library of treatments for patients against which their cancer has been tested. Explant cultures can also be used for other cytotoxicity assays (such as LDH or MTS) through their measurement of their activity in the conditioned media (120–122).

Historically however there have been issues with the short duration of cell viability [likely as a consequence of absent functional vasculature (123)] and the lack of standardised and comparable readouts. Explants generally require a reasonable amount of tumour sample and are not self-renewing like PDOs. This means they only represent the drug sensitivity and resistance at a single moment in time and any subsequent drug sensitivity and resistance testing (DSRT) to the original treatment would have to be carried out on a fresh biopsy sample.

However new advances in the field of explant technology are being developed, some of which have shown promising results with good clinical correlation, such as the CANscript and Curesponse™ platforms (124, 125). These have demonstrated explants to be a fast, reliable platform for drug testing that accurately represents *in vivo* tumour response. Explants have also demonstrated the ability to evaluate underlying treatment resistance mechanisms (126). Both models use tumour explants or slices from fresh tumours that have been sliced and cultured in a media or matrix in order to obtain a quantitative score following drug treatment based on various parameters (such as pathological and morphological analysis, cell proliferation, cell viability and cell death). Such scores can then predict response to commonly used therapeutics and have shown good sensitivity and specificity in solid tumours (124, 125). A clinical trial further evaluating the sensitivity and specificity of the Curesponse™ model [*Ex-Vivo Organ Culture (EVOC)*] is due to open soon (NCT04599608), with a view to progression to a subsequent phase II trial of EVOC predicted therapy versus physician choice if successful. If this accurately reflects *in vivo* tumour dynamics, this could further progress the next generation of personalised medicine.

Other techniques have utilised dynamic, agitation-based culture systems to improve explant perfusion and thus viability (127), enabling thicker slices to be cut comprising more of the TME. Such methods also increase explant longevity. Extensive validation of these methods as well as direct comparison with other cancer models is awaited.

#### 3.2.5 Organotypic Omental Models

Given the omentum is a common site for ovarian cancer metastases, 3D models have been developed attempting to recreate the omental microenvironment present *in vivo* to further understanding of cancer adhesion and invasion, as well as provide models for high throughput drug screening (128). This model involves use of human primary mesothelial cells and fibroblasts from healthy omentum obtained during surgery, embedded in an extracellular matrix of collagen. Primary ovarian cancer cells or immortalised ovarian cell lines are then added to recreate the metastatic niche (128).

This has provided a useful tool for understanding the mechanistic processes underlying early ovarian cancer metastasis (129–132) as well as the important role that omental mesothelial cells and fibroblasts play in this process (128). High throughput drug screening with this method has been shown to correlate with *in vivo* efficacy in mouse models (133, 134) as well as highlighting the discrepancy between 2D and 3D models for drug screening and thus the importance of inclusion of TME components. This model has also subsequently been used to identify novel compounds as potential future therapeutic strategies (135). However it is important to note that this model does not contain other aspects of the TME such as vasculature, endothelial cells, and immune cells, and as with other 3D models relies on an artificial ECM (136).

A multi-cellular omental model has been recently developed incorporating 4 different cell types (HGSOC cell lines, primary human mesothelial cells, adipocytes and fibroblasts) which has dissected the role platelets play in HGSOC metastases, and is also likely to permit further evaluation of this metastatic niche (137). Other attempts to incorporate more of the TME in 3D models include the production of a hybrid 3D system (in this case in breast cancer) that comprises mammary epithelial cells in conjunction with fibroblasts and endothelial cells embedded on a porous scaffold (138). This method may help better reflect *in vivo* angiogenesis and thus improve on recreating tumour-stroma interactions. However widespread use of this model for high throughput drug screening has not yet been attempted.

## 4 Research Potential of Pre-Clinical 3D Models for Biomarker and Therapeutic Evaluation

The large volume of pre-clinical models available for ovarian cancer have already yielded significant diagnostic and therapeutic advances. However, despite this, high relapse rates remain for patients with advanced HGSOC, with treatment resistance a common occurrence and thus there is a significant unmet need and demand for improvement. Out of the current available models, PDOs have been recognised to have significant potential for further investigation of EOC behaviour and resistance due to their organ specificity, high concordance with *in vivo* tumour genomics and applicability for drug testing (10, 96, 98). Tumour explant models, although they lack the ability for extended passaging, permit immediate low to high throughput drug screening on an individualised level which is likely to contribute to further advances in personalised therapeutics for patients.

### 4.1 3D Models Retain Features of Genomic Heterogeneity

A significant challenge with HGSOC treatment is the intrinsic genetic instability and diversity present. For a research platform to be effective and representative of the *in vivo* tumour, it is important that the models used reflect this genomic instability and intra-tumoural heterogeneity. Previous studies using PDOs in other cancers (139, 140) have highlighted the concerns of specific tumour clone selection *ex vivo* as a result of the growth factors required for PDO production, as well as the time required for generation.

Short-term patient derived HGSOC organoid cultures (7-10 days *ex vivo* growth) have been shown to have high morphological, molecular and genetic homology with the original tumour (96). More specifically, a median of 98.2% of mutations that had been identified in the primary tumours were found in the matched tumour organoid line, and 98.8% of mutations observed in the organoid lines were also present in the parent tumour (96). Similar genomic allelic imbalances and CNAs were also observed between parent tumours and PDOs. Whole exome sequencing (WES) did not show a significant accumulation of somatic mutations in the PDOs, nor did early cultures exhibit changes in driver mutations. However, this study only assessed short-term PDO development rather than long-term models. Interestingly this work showed that BRCA1/2 or Fanconi anaemia pathway mutations were not pre-requisites or indeed required for HR deficiency and PARPi sensitivity (96). Additionally, it highlighted the importance of stalled replication fork protection defects in treatment sensitivity and response. This work also demonstrated that PDOs derived from patient tumours, in this case collected at recurrence, can reflect the genomic intra-tumoural heterogeneity exhibited in disseminated HGSOC (96).

Generation of long term, stable HGSOC PDOs has been more challenging. This has been achieved with varying degrees of success in a number of studies (10, 98, 100, 101), although consistently EOC PDOs have been shown to accurately represent the histology and intra-tumoural heterogeneity of the original tumour tissue. This included one study where PDOs were passaged over 30 times with whole genome sequencing showing maintained CNAs (98). DNA methylation analysis showed similarly that the epigenetic profile was maintained in PDOs even after extended passaging. However, accumulation of mutations with generation of an “*in vitro*” phenotype remains a concern with TP53 loss of heterozygosity observed and also new somatic nucleotide variants emerging with increased passaging (98–100).

In a study by Nanki et al., seven PDO lines were generated from different EOC subtypes (HGSOC, clear cell, endometrioid) with success rates of 80% (10). Targeted capture sequencing of 1,053 cancer-related genes demonstrated that 59.5% of the primary tumour genomic characteristics were shared between primary tumours and PDOs which is lower than that seen with previous studies (96); this was thought to be due to the fact the sequencing was performed after a higher number of passages compared to the Hill et al. study. Importantly key DNA variants for tumorigenesis such as BRCA1/2, MLH1, TP53, ARID1A and PIK3CA were all maintained. 26.7% of the variants identified were seen solely in the tumour and 13.8% were identified only in the organoid lines (10), implying a degree of *in vitro* selection as seen with other studies. Indeed, some wild type alleles such as RB1 were noted to have been lost during PDO development. However overall variant allele frequency and CNAs were similar between primary tumours and PDOs.

### 4.2 Models for Evaluation of Predictive Biomarkers

Predictive biomarkers of treatment response are very valuable to assess clinical benefit in patients from treatments and remains an unmet need in ovarian cancer.

Most patients with BRCA1/2 mutations frequently respond well to platinum-based chemotherapy and PARP inhibition, however some patients will develop early resistance (141, 142). Cancers which demonstrate similarities in drug response to BRCA mutant cancers, frequently possessing mutations in other homologous recombination genes, are said to exhibit a “BRCAness” phenotype. The presence of mutations in one or more homologous recombination genes (whether BRCA1/2 or otherwise) has been shown to predict responsiveness to platinum based chemotherapy (9) and PARPi (57, 58). Different methods for evaluating HRD have been developed, which can assess germline or somatic mutations in HR repair genes, or measure the genomic scar or signature that has occurred as a consequence of HRD [calculated on the extent of loss of heterozygosity, SNVs, insertion-deletion mutations and telomeric allelic imbalance (e.g. myChoice^®^ CDx, Myriad Genetics)] (96, 143). More recent techniques have looked at the proficiency of HR by virtue of the assembly of RAD51 foci at sites of DNA damage such as the Repair Capacity (RECAP) test (144, 145). This test, initially developed to assess HRD in breast cancer, was validated using tumour slices and has demonstrated utility now in ovarian cancer (144), highlighting the flexibility and utility of tumour slices, and the potential extent of their use as both a diagnostic as well as therapeutic biomarker. Functional HR status has also been assessed in PDOs using the RECAP test and has been shown to correlate with PDO drug sensitivity (e.g. platinum chemotherapy, PARPi) (10, 98) and also with clinical response (96). However, as observed with *in vivo* studies, merely the presence of HRD did not guarantee platinum or PARPi responsiveness (96) indicating greater complexity warranting further investigation.

Whilst a number of different prognostic and predictive biomarkers for ovarian cancer have been explored (146), sensitive and specific biomarkers that could permit early stage diagnosis, or else that could clearly delineate treatment responders from non-responders are still awaited. However, the availability of 3D models, such as explants and PDOs, that accurately represent *in vivo* tumour is anticipated to further development in this field.

### 4.3 3D EOC Models of Drug Sensitivity and Resistance

The heterogeneity in ovarian cancer genotypes and phenotypes, as well as differential patient responses to treatment means the ability to reliably test different drugs *ex vivo* in a way that accurately depicts the *in vivo* environment is a key focus in drug development.

EOC PDOs have undergone drug sensitivity and resistance testing (DSRT) in a number of different studies. Short-term PDOs have been tested against different compounds including olaparib, carboplatin, gemcitabine, paclitaxel, doxorubicin, the CHK1 inhibitor prexasertib and ATR inhibitor VE-822, with correlations observed between PDO resistance/sensitivity and *in vivo* response (96). Additionally, correlations between PARPi resistance and restoration of HR function were seen, consistent with PARPi resistance clinically (96). This study also highlighted the correlation between replication fork instability and carboplatin, prexasertib and VE-822 sensitivity, and that CHK1 inhibition in combination with carboplatin or gemcitabine could promote replication fork instability, providing a rationale for potential combination of these agents clinically.

Kopper et al. also showed that PDOs can be used for DSRT, including for different histological subtypes (98). They demonstrated variable sensitivity to commonly used chemotherapies (carboplatin and paclitaxel) amongst other drug treatments (98). Lines were also tested for PARPi sensitivity, and this was shown to correlate with HRD (98). Additional studies have been performed by different groups to assess organoid sensitivity, for example Maenhoudt et al. treated EOC PDOs with a range of conventional chemotherapy agents (carboplatin, paclitaxel, doxorubicin, gemcitabine) and demonstrated heterogeneity in responses between different PDO lines, though without *in vivo* comparison (101). Nanki et al. used 23 FDA approved drugs for PDO DSRT and noted clinical correlations between PDOs and *in vivo* responses (10). Maru et al. used organoid-derived spheroids to demonstrate responses to carboplatin and paclitaxel, though did not perform any *in vivo* comparisons with patient response data (100).

Whilst previous EOC PDO–clinical correlations were largely anecdotal, more formal analyses have demonstrated statistical significance between organoid response to treatments and clinical response, as determined by histological, radiological and biochemical markers (99). Interestingly in this study intra-patient drug response heterogeneity was also seen in a small number of PDO lines derived from multiple tumour samples from the same patient. In some cases, this correlated to genetic differences, but in other cases no clear cause for this drug response heterogeneity could be found (99).

In addition to treatment response, PDOs have now been used to further study treatment resistance in EOC. Cisplatin-sensitive and cisplatin-resistant organoid lines were established, with RNA sequencing confirming that upregulation of the serine/threonine kinase Aurora-A conveyed cisplatin resistance (147). This was shown to be *via* the SOX8/FOXK1 pathway resulting in suppression of cell senescence and increased glycolysis. This work could uncover new therapeutic avenues to explore in the frequent setting of treatment resistance in HGSOC treatment.

*Ex vivo* explant cultures have also been established as models for DSRT in a number of different cancers (148–150) including EOC (127, 151). Ricciardelli et al. used cryopreserved ovarian cancer tissue for their model and cultured predominantly EOC explants *ex vivo* on a gelatine sponge (151). They demonstrated preserved histological features in the cryopreserved tissue (compared to freshly fixed tissue) in addition to preserved tissue architecture and B-lymphocyte populations (as determined by CD45 immunohistochemistry) which was retained following 72 hours of culture. They also observed differential carboplatin sensitivities in platinum resistant/sensitive tumours, as well as demonstrating an increase in apoptosis when carboplatin was combined with a hyaluronan inhibitor (not currently in clinical use for ovarian cancer) (151).

Abreu et al. used a novel agitation-based culture to prolong tissue viability of fresh tumour explants of different ovarian cancer histologies, demonstrating similar histological features following prolonged culture (up to 30 days) as well as retained CD4 and CD8 T cell populations (127). Similarly, the ratio of epithelial cells to fibroblasts was also retained following culture. They also demonstrated response to commonly used chemotherapeutics, as indicated by reazurin reduction capacity. These studies highlight the strengths of explant cultures, notably their ability to retain tissue architecture and cell populations (i.e., the tumour microenvironment) on which DSRT can be performed.

### 4.4 Limitations to Organoid and Explant Cultures

Although PDOs have shown to accurately model tumour growth, they are largely composed of neoplastic epithelium, and lack a fully accurate representation of *in vivo* stroma, vasculature and immune cells, which play an important role in tumour growth and dissemination. The tumour microenvironment is an important facilitator in tumour growth and treatment response. Additionally, the availability of resources such as nutrients and oxygen contribute to intra-tumoural heterogeneity (152, 153). Thus, whilst they possess good 3D architecture, PDX models or humanized mice are considered to be better for assessing stromal or immune interactions than PDOs (154).

As mentioned in studies discussed above, the derivation rate of organoids is variable which could limit clinical applicability. There is also batch-to-batch variability in Matrigel or similar BMEs’ composition (155). Matrigel is produced from mouse tumour lines which could hamper *in vivo* comparisons, and also impair drug penetration with subsequent detrimental effects on the utility of organoids for DSRT (156). In addition, human organoids can display more varied growth compared to murine organoids that correlates to the grade of the tumour histology, as well as the condition of the tumour biopsy (157). This can lead to differential amounts of necrosis present and thus variable correlations with *in vivo* treatment response.

Explants are limited by their short duration of cell viability [although methods such as the agitation-based culture system by Abreu et al. are trying to circumvent this (127)]. A reasonable quantity of tumour sample is generally required for generation of explant cultures, and they are not self-renewing like PDOs, so the explant cultures only represent a snapshot of a tumour at one timepoint. Practical complications in slicing can also arise as a result of inherent tumour heterogeneity and slicing and cultivation itself can also induce changes in biomarker expression and stress pathways (117). A full representation of the advantages and disadvantages of different tissue models is shown in **Table 1**.

## 5 Novel Technologies in 3D Models

### 5.1 Advances in Organoid Technology

#### 5.1.1 Microfluidic Based Models

Ascitic fluid accumulation subjects ovarian cancer cells to shear stress which has been shown to affect ovarian cancer progression through increased epithelial-mesenchymal transition (158, 159), expansion of cancer stem cells (159) and peritoneal spreading (160). Microfluidic models that could mimic the *in vivo* environment have thus generated significant interest. Microfluidic devices (tumour-on-chip devices) may only require small amounts of tissue (~100,000 cells, comparable to that obtained from a small fine needle aspirate) and reagents (161) and they can also incorporate stroma (162, 163), vascular (164) and immune components (165, 166). As such they are considered to potentially possess better spatial organisation of the TME than other 3D models.

Microfluidic based spheroid models can be generated in different ways including *via* the hanging drop method (167), droplet generation devices (168), hydrodynamic traps (169) or microwells (161, 170). 3D models have now been developed using ovarian cancer spheroids (161, 171) that theoretically generate a more physiological environment for ovarian cancer cells undergoing metastasis. In particular, spheroids grown from PDX ovarian cancer murine models in a microfluidic platform were shown to be superior to those grown in Matrigel or standard culture in terms of spheroid yield, uniformity and viability, phenotypic gene expression and proliferation (161).

However there are limitations such as the inability to culture healthy epithelium thereby preventing control comparison or the diffusion of nutrients limiting spheroid size (172). Additionally, there are limited possibilities for *in situ* probing of the dynamic changes occurring within the ECM as microfluidic systems are often in a closed set up (173).

#### 5.1.2 Alternative Extracellular Matrices

Micro-patterned 3D tumour platforms use natural or synthetic hydrogels in micro-engineered models to further investigate the interactions within the TME (173). These techniques allow generation of spatially organised cellular constructs that permit localisation of target cells (e.g., fibroblasts) for study of their role in tumorigenesis (174, 175). They also permit manipulation of matrix stiffness within the model, which is not possible with conventional Matrigel or similar basement membrane extract matrices (175, 176), whilst avoiding the batch variability and undefined composition associated with Matrigel. However, compared to natural hydrogels, synthetic hydrogels require supplementation with factors that upregulate certain cellular process e.g. growth and adhesion (177).

Synthetic hydrogel models have demonstrated effective co-culture of different TME components to enable intercellular network formation (176). In this particular model, hydrogels were engineered with different peptide/protein combinations in order to identify the optimum combination for ovarian cancer spheroid growth, as well as that of human umbilical vein endothelial cells and human mesenchymal stem cells. This study highlighted the versatility of such methods, although issues with scalability and cost need to be resolved. Different hydrogels have also been explored [e.g. polyethylene glycol (178) or Gelatine methacrylamide-based hydrogels (179)] which have shown comparable results to Matrigel. Drug treatment response assays have also been performed (176, 179) showing the different applications of these matrices, though they are not yet in widespread use.

Decellularized extracellular matrices (ECMs) have also recently been investigated as a 3D model for cancer, following their use in tissue engineering and tissue regeneration (180). Decellularized ECMs can be generated from both healthy and malignant tissue and have been shown to maintain many of the cytokines and growth factors that were present in the original tissue (181). Decellularized ECMs have demonstrated utility as a clinical model in colorectal, lung and breast cancer (182–184) although as yet are not in widespread use and to our knowledge have not been evaluated in EOC.

#### 5.1.3 *In Vitro* Incorporation of Functioning TME

Given its important clinical implications, there have been attempts to generate an *in vitro* immune interaction with organoids. Jenkins et al. showed that the PD-1/PDL-1 interaction could be simulated in spheroid models of different solid tumours (185), though this was lacking tumour immune specificity. Co-culture of tumour infiltrating lymphocytes (TILs) with neoplastic epithelium organoids is however difficult, although in mismatch repair deficient colorectal cancer and non-small cell lung cancer, tumour organoids have been co-cultured with peripheral blood lymphocytes as a way of enrichment for tumour-reactive T cells (186). A different PDO model with an air-liquid interface has shown the ability to maintain the tumour-immune microenvironment, notably PDL-1/PD-1 interactions (157). This model, using melanoma, renal cell carcinoma and non-small cell lung cancers, was shown to preserve neoplastic epithelium with endogenous immune and stromal components, thereby preserving the TIL/tumour cell interaction. Whether this organoid model can be modified to incorporate peripheral immune components as in other studies (186) remains to be seen.

This is significant clinically for the investigation of tumour sensitivity and resistance to checkpoint inhibitors as well as the ability to produce patient specific T cell cultures (theoretically for adoptive T cell transfer). However, these studies were predominantly demonstrated in tumour types with a high mutational burden and thus it may not be feasible to perform in cancers like OC which typically is associated with a low mutational burden. Whilst it may not have a high mutational burden, EOC is viewed as potentially immunoreactive given the association seen between clinical outcome and the presence of tumour infiltrating lymphocytes (187). Strategies to convert tumours that are immune-desert or immune-excluded into immune-inflamed phenotypes that respond to immunotherapy have been proposed (188). However so far immunotherapy has not yielded significant benefit in OC in randomised phase III trials (189). 3D models that can reliably simulate interactions within the TME are therefore a critical unmet need.

Another study focused on functional and single-cell RNA sequencing profiling on HGSOC organoid-immune cell co-cultures and identified 3 immune therapy targets in HGSOC through use of a novel bi-specific antibody (190). This led to subsequent identification of 2 new potential immune therapies for HGSOC (a BRD1 inhibitor and a bi-specific anti-PD-1/PD-L1 antibody) although these are yet to be tested clinically.

Other components of the TME have been incorporated into co-cultures with EOC 3D models such as fibroblasts (191, 192). Use of normal fibroblasts in co-culture with ovarian cancer spheroids has been used as a model for *in vitro* epithelial-mesenchymal transition (192), a key step in tumour growth and metastasis. Furthermore, the presence of fibroblasts in co-culture with EOC cells resulted in an upregulation of genes that have been associated with tumour formation, angiogenesis and metastasis (191). Whilst there was no difference seen in the chemosensitivity between 3D mono and co-cultures in this study (191), these studies provide an insight into the supportive role of fibroblasts in tumour growth and further studies of 3D EOC co-cultures are warranted. As discussed earlier, other co-cultures have also incorporated adipocytes in addition to normal fibroblasts as part of an omental model (137). This study highlighted the role that platelets play in the production of particular ECM components that portend an adverse prognosis, as well as their role in driving malignant invasion. These co-culture methods are likely to aid re-creation of the primary and metastatic niche *ex vivo* which will hopefully lead to therapeutic advances.

### 5.2 Additional Applications of 3D Models

Manipulation of fallopian tube organoids with a lentiviral gene vector or CRISPR/Cas9 mutagenesis has already been used to generate genotypically different murine HGSOC organoids for drug testing (193). Subsequent drug testing revealed a chemotherapy/immunotherapy combination that generated durable T cell responses in TP53-/-, CCNE1/AKT2 overexpressed and KRAS mutant organoid lines but not in other lines, thereby highlighting the potential clinical utility of PDOs in patient drug development and the evaluation of potential predictive biomarkers.

PDOs have also recently been used to investigate the site of origin of HGSOC given the differing hypotheses of HGSOC originating from fallopian tube or ovarian surface epithelium. It was previously shown that PDOs could be grown from human fallopian tube epithelial cells, and that stemness and differentiation was dependent on the Wnt and Notch signalling pathways (194, 195). Subsequently CRISPR-Cas9 genome editing was used to introduce mutations in commonly mutated HGSOC genes (e.g., TP53, BRCA1, PTEN, NF-1) into murine organoids (196). Both ovarian surface and oviductal (fallopian tube) epithelium were shown to produce high-grade ovarian tumours, although oviductal organoids expanded faster and had higher tumorigenic potential on transplantation into immunodeficient mice. Importantly from a clinical perspective, the two types of epithelia showed differential drug responses (196). Similar results showing the dualistic origin of HGSOC were also observed in other murine organoid models following knockout of TP53 and Rb as well as differential chemosensitivities of fallopian tube *vs* ovarian surface epithelium (197).

## 6 Future Clinical Perspectives

For future clinical implementation of these 3D model systems to simulate *in vivo* drug responses, it is imperative that these models are accurately correlated with *in vivo* responses. Furthermore, for uptake as drug testing platforms, they must operate with high sensitivity and specificity with robust, reliable assay read-outs. From a PDO perspective, there is likely to be more manipulation of organoids to re-create clinical scenarios such as treatment resistance as a way of exploring new therapies or combinations before use in the clinic. Additionally, ways of better recreating the TME *ex vivo* are likely to be researched given the significant *in vivo* importance of the TME.

In addition to the clinical perspective, there is also a research and economic perspective in that the extensive cost involved in early phase drug testing could be significantly mitigated with more accurate and clinically relevant *in vitro* models, with fewer patients exposed to unnecessary toxicity of early phase trials if their treatment was unlikely to be beneficial. 3D models could also reduce the use of mouse models, with ethical, economic and practical benefits. Many early phase trials are now a basket design where patients are grouped and then subsequently treated with a variety of different compounds based on particular biomarkers, and *in vitro* testing pre-treatment could help identify which patients are most likely to derive benefit from particular treatments. Whilst there is significant clinical utility amongst EOC and other common cancers, this technology is also likely be very helpful for rare tumours as low numbers frequently hinder clinical trial development given the significant numbers required to recruit. There are already a number of different clinical trials that are exploring the utility of PDO as models for patients’ treatments (e.g., NCT04555473, NCT04768270) and other tumour explant trials that are soon to open (e.g., NCT04599608). This is likely to be where the majority of early phase trials are heading, with benefits for patients, clinicians, researchers and pharmaceutical companies alike. Whether PDOs could be used in combination with explants to give the most representative *ex vivo* simulation of *in vivo* response remains to be evaluated.

## 7 Summary

Overall, there has been significant progress in the development of new *ex vivo* models in recent years which is likely to significantly improve research into novel biomarkers and therapeutics. They also have the potential to change the way both academic research and drug development are conducted, whilst also revolutionising patient treatment pathways to incorporate a new level of personalisation into the clinic to optimise patient outcomes.

PDOs have been an exciting development that are likely to play an increasing role in biomarker and therapy development. The absence of accurate representation of the TME at present hinders evaluation of an important part of the tumour-treatment response and there remains a need to improve models to reliably represent *in vivo* therapeutic efficacy. Whilst tumour explants provide a better representation of the TME *ex vivo*, their use also has its limitations, notably their short duration of *ex vivo* viability and the absence of self-renewing capability.

However, with both commercial and clinical interest focused on refinement of 3D models, this is likely to be the forefront of the next generation of individualised treatment assays as well as contributing to drug development to improve prognoses and treatment options for this devastating disease.

## Author Contributions

JC and PC were involved in the initial review design and composition. JC, PC, CF, and JK contributed to manuscript content and format. All authors contributed to the article and approved the submitted version.

## Funding

JC is supported by the Dangoor Fellowship. PC and CF acknowledge support from the Ovarian Fund, Imperial Health Charity.

## Conflict of Interest

The authors declare that the research was conducted in the absence of any commercial or financial relationships that could be construed as a potential conflict of interest.

## Publisher’s Note

All claims expressed in this article are solely those of the authors and do not necessarily represent those of their affiliated organizations, or those of the publisher, the editors and the reviewers. Any product that may be evaluated in this article, or claim that may be made by its manufacturer, is not guaranteed or endorsed by the publisher.
